# The experiences of young people, parents and professionals of using the attend anywhere video consultation system in a child and adolescent mental health service: a mixed-methods approach

**DOI:** 10.3389/frcha.2023.1194302

**Published:** 2023-08-11

**Authors:** Emer Gormley, Ruth Melia, Sharon McCormack, Bonita Paige Phayer, Jessica Madden

**Affiliations:** ^1^ School of Psychology, University of Galway, Galway, County Galway; ^2^ Psychology Department, University of Limerick, Limerick, Ireland; ^3^ Child and Adolescent Mental Health Services, HSE Mid-West, Naas, Ireland

**Keywords:** video enabled care, attend anywhere, digital mental health, child and adolescent mental health (CAMH), eHealth

## Abstract

**Background:**

In 2020, Attend Anywhere video consultation service was introduced across the Irish public health service to facilitate the provision of health interventions remotely in light of COVID-19-related restrictions. This study aims to explore the experiences of young people, their parents and their clinicians, of using the newly introduced Attend Anywhere video consultation as part of their Child and Adolescent Mental Health Service (CAMHS).

**Method:**

A cross-section of twenty-nine young people, their parents and clinicians working in CAMHS Clare completed a survey pertaining to their experiences of using Attend Anywhere as part of their service. A cross-disciplinary research steering group of CAMHS clinicians adapted the NHS Scotland evaluation of Attend Anywhere / Near Me survey to better capture experiences in a CAMHS setting. The survey included both quantitative and qualitative items. Descriptive statistics were used to examine quantitative data. Qualitative data was analysed using Thematic Analysis.

**Results/Findings:**

Results demonstrated a decrease in the number of barriers reported by clients and professionals in accessing the CAMH service following the introduction of Attend Anywhere video consultation. Overall, the majority of professionals reported that they would use Attend Anywhere again, whereas almost a quarter of clients reported that they did not wish to use it again. Clients indicated a preference for receiving face-to-face services over other service provision options and this finding was associated with not having to rely on technology or manage connectivity issues and finding it easier to build the therapeutic relationship in-person.

**Conclusion:**

Findings suggest that both professionals and clients value face-to-face service provision while also acknowledging the benefits of Video Enabled Care in overcoming access barriers. We conclude that VEC be offered as an option in a blended service model, in conjunction with rather than as a replacement of face-to-face service provision.

## Background

There have been significant and recent strategic drivers for the integration of digital technology, and video conferencing services in particular in Health Services. In Ireland, Slaintecare (1) aims to transform health and social care services over a 10-year period, espousing the need for an integrated model of care, including the use of eHealth. In 2020, Attend Anywhere (video consultation service) was introduced across the health service to facilitate the provision of healthcare intervention remotely in light of COVID-19-related restrictions. The service was piloted extensively in Child and Adolescent Mental Health Services in HSE Mid-West due to pre-existing clinical space shortages, the need to improve access for those living in remote areas, and to facilitate social distancing on-site.

Recent research has demonstrated positive outcomes and experiences for clients and professionals of using video consultation during the COVID-19 pandemic (2–7). Studies have concluded that the main benefit of video consultations has been the ability to provide a safe, accessible alternative to face-to-face appointments. Rather than suggesting that video consultations should replace in-person appointments, research indicates the need for video consultations as a means of providing a flexible service. Video-enabled care was primarily introduced within the Irish public health system in response to the restrictions enforced during the COVID-19 pandemic. A report on the findings of the first national evaluation of the use of video enabled care (VEC) across the health service in Ireland (8) reported on survey responses from 696 patients and 719 Health Care Professionals (HCPs) across healthcare settings during the COVID-19 pandemic. Satisfaction levels with VEC were high overall with patients reporting higher levels of satisfaction than HCPs. Attend Anywhere was the most widely used platform by healthcare professionals (51.5%) with 14.1% using Cisco Webex, 8.4% Blue Eye, 6.4% MS Teams, 6% Zoom, 2.0% WhatsApp and 10.7% using “other” platform not specified.

A common difficulty of using video consultations highlighted across studies was technological issues (1, 3, 9). Dissatisfaction with VEC was largely related to technical problems or the reported appropriateness of VEC for the purpose of the consultation. It has been argued that the rapid move to video enabled care exposed the “digital divide” as a social determinant of health. Families of lower socioeconomic status, and those experiencing language barriers were less likely to have access to technology, broadband internet, and digital literacy to effectively obtain care through video-based appointments (10). Such research indicated that it may be necessary to offer alternatives to video visits, including in-person visits or telephone-only visits, to avoid exacerbating the health care disparities experienced. Ethical concerns have also been raised regarding the use of technology in mental health service provision, particularly in relation to privacy and data security, monitoring and assessing risks, and inconsistent practices in the inclusion of end users in development and design (11). The findings of a scoping review suggest that a shift toward patient-centred design is particularly necessary for digital mental health technologies for younger people as the needs of younger people are typically under-addressed (12).

In 2020, Wherton and Greenhalgh, published an evaluation of the use of Attend Anywhere in NHS Scotland. The evaluation studied the use of this platform across clinical specialities, departments, and levels of care, utilizing interviews with professionals, adult patients, and carer or relatives to collect data. The current study therefore aims to extend our knowledge of VEC by exploring the experiences of young people, parents and professionals of using Attend Anywhere within a CAMHS setting specifically.

The integration of digital technology into routine mental health care arguably constitutes a significant change in service provision and design which is likely to greatly affect the lives of young people. The participation of children and young people is fundamental to a child-centred, rights-based approach and aligns with the Lundy Model of Youth Participation. The Lundy model (13) comprises four chronological steps in the realisation of a child's right to participate. First, “space”: children must be provided with the opportunity to express a view in a space that is safe and inclusive. Second, “voice”: children must be facilitated to express their view. Third, “audience”: the view must be listened to. Fourth, “influence”: the view must be acted upon, as appropriate. Mental health service professionals within the Irish public health system are guided by the “National Strategy on Children and Young People's Participation in Decision-Making 2015–2020′ and “Toward the Development of a Participation Strategy” (14), documents that set out a roadmap for Good participatory practice and support the process of actively engaging young people and their parents, in such a process.

### Aim of current study

To explore the experiences of young people, their parents and their clinicians of using the newly introduced Attend Anywhere video consultation in CAMHS Clare.

### Research questions

1. What are the barriers to and benefits of attending face-to-face CAMHS services?
2. What are the barriers to and benefits of using Attend Anywhere as part of the CAMHS service?
3. How willing are clients and professionals to continue using Attend Anywhere?
4. How would clients and professionals like to receive/provide their CAMHS service going forward?

## Method

### Study design

The current study represents a cross-sectional, exploratory design in a clinical setting. The study utilized a mixed-method approach to include both qualitative and quantitative data. Data was collected between November 2020 and March 2021, during the COVID-19 pandemic when restrictions were in place.

### Setting

#### Use of attend anywhere

At the time of data collection, Attend Anywhere was being used across CAMHS Clare and all participants were recruited from that service. Child and Adolescent Mental Health Services are secondary level mental health services accessed by children and adolescents aged 6–18 years experiencing moderate to severe mental health difficulties. CAMHS Clare provides services to children and young people requiring secondary level mental health services residing within their catchment area—county Clare.

At the time of study enrolment, three different service models were in use:

Hub-home: Clinician connects from clinic to patient at home.
Dyadic hub-spoke: Clinician in specialist “hub” centre connects to patient in remote “spoke” health or care site without additional staff member present (e.g. hospital bed).
Triadic hub-spoke: Clinician in specialist “hub” centre connects to patient in remote “spoke” health or care site with an additional staff member [nurse, GP, healthcare support worker (HSW)] present.

In all of the above models, the specialist clinician occasionally consulted from home (while self-isolating/cocooning). The model primarily used in CAMHS Clare was the Hub-home model which involved the young person accessing Attend Anywhere while at home and the clinician based in the specialist hub (the CAMHS clinic). On occasion, for example while a client was admitted to hospital, the dyadic hub-spoke model was used for short periods of time. When restrictions allowed in-person school attendance, and where clinically appropriate, a small number of clients accessed their CAMHS appointment from school, with the support of their school counsellor or other member of school staff. While these models were in operation at the time of the study, the primary model for all participants in the course of the current study was the hub-home model.

#### Participants

A cross-sectional study of twenty-nine young people, their parents and clinical professionals using Attend Anywhere within CAMHS Clare were selected for inclusion in this study (*N* = 29). Participants were included if they met the inclusion criteria: young people, parents or professionals working with young people who (i) were accessing CAMHS Clare, (ii) had utilized Attend Anywhere as part of that service at least once, and (iii) consented to taking part. Participants were excluded if they did not meet the above inclusion criteria. Initially, 30 out of the 61 participants invited to take part had responded, however one participant was excluded from the analysis as their responses indicated that they did not have any experience of using the Attend Anywhere platform.

Frequencies of type of responder for our sample are summarized in Table 1. Frequencies show there were 17 young people and their parents (clients: 58.6%) and 12 CAMHS clinicians (professionals: 41.4%). Of the 17 participants identified as clients, 9 were young people and 8 were the parents of the young people who completed the survey. While demographic information was not gathered in the course of the study, the service was designed for use by children and young people aged 6–18 years. The age range of clinicians working in the service was 25–65 years.

**Table 1:** Frequencies of responders.

	*N*	%
Clients	17	58.6
Professionals	12	41.4

#### Measures

A mixed-method survey (Supplementary Appendix S1) was used to gather both quantitative and qualitative data pertaining to participant's experiences of using Attend Anywhere in CAMHS Clare. The measure used was informed by the Wherton and Greenhalgh (15) tool developed as part of the NHS Scotland evaluation. As the Wherton and Greenhalgh measure was designed for use within the broader health system, the research team reviewed and developed the current survey to reflect the experiences of participants accessing a child and adolescent mental health service setting specifically.

#### Procedure

A cross-sectional study of young people, parents and professionals using AA as part of CAMHS service provision was conducted. Data was collected at one time-point and clients, parents, and professionals, were invited to complete a survey exploring their experiences of using Attend Anywhere as part of service provision. CAMHS professionals who had experience of using Attend Anywhere with clients were informed of the study in-person at team meetings and invited to take part. Client participants were recruited through their CAMHS keyworkers who identified clients who utilized Attend Anywhere as part of their CAMHS service provision. A key worker is a client's point of contact on the mental health team responsible for helping to co-ordinate the client's care. As a central database detailing Attend Anywhere use by individual clients as part of service provision was not available, researchers required CAMHS keyworkers to identify clients with whom AA had been used to facilitate recruitment.

Potential participants were invited by letter to consent to and complete a survey via an online secure platform or by post should they wish to take part (see Supplementary Appendix S2). The information sheet provided participants with information on informed consent and their right to withdraw from the study. Parents were asked for written consent for their young person to take part and the young persons were asked for written assent. Survey responses were anonymous to address the risk of bias.

##### Statistical analysis

The quantitative data was analysed using IBM SPSS Statistics 27 software. There was no missing data. Descriptive statistics were utilized to present the findings from the quantitative data. Exploratory t-tests were used to establish if there were any differences between groups (i.e., clients and professionals) in terms of using Attend Anywhere again in the future.

#### Qualitative data analysis

Due to the exploratory nature of this study with a relatively under-researched sample, a phenomenological approach was deemed most suited. The qualitative data was transcribed and Thematic Analysis used to identify themes. Thematic analysis is the process of identifying patterns or themes within qualitative data. It is a method rather than a Methodology (16) which means that it is not tied to a particular epistemological approach or theoretical perspective. This bestows the advantage of flexibility on this method. The researchers followed Braun and Clarke's (16) 6-step framework: become familiar with the data, generate initial codes, search for themes, review themes, define themes, write-up.

##### Ethical considerations

Ethical approval was obtained from University Hospital Limerick Research Ethics Committee before data collection commenced, and it was performed in accordance with the 1964 Helsinki declaration and later amendments. Data was collected and stored in line with relevant HSE General Data Protection Regulation (GDPR) guidelines and the Data Protection Acts 1988–2018. No potential harm from the study was identified for participants.

## Results

### Statistical strategy

This study explored client and professional's experiences of using Attend Anywhere in the CAMHS Clare service. Data was gathered using a multiple-choice survey (as outlined above, see Method section) and analysed using IBM SPSS v27 Statistics software.

### Screening

Raw data was inputted to IBM SPSS Statistics v27 software. Data was cleaned and screened in line with Pallant (17) guidelines. No errors or outliers were found for any of the variables and therefore no responses were excluded. There was no missing data in the data set.

### Preliminary analyses

Preliminary analyses were carried out in order to explore and describe the characteristics of the sample and the variables used to check the assumptions that are necessary for running an independent t-test and to determine whether parametric or non-parametric analyses would be used. The “ I would use Attend Anywhere again” variable was assessed for normality and scatterplots graphed (Supplementary Appendix S3).

### Descriptive statistics

Overall, the response rate for this study was 48%, that is, 29 of the 61 individuals invited to take part completed the survey and their data was included in the current analysis. Initially 30 participants responded but on further inspection, the individual's responses indicated that they had not used Attend Anywhere and were therefore ineligible to take part, their responses were not included and are not reported on here. 59% of professionals or 12 of the 20 professionals invited to take part completed the survey. 41% of clients or 17 of the 41 clients invited to take part completed the survey. 17 clients (young people and their parents), and 12 CAMHS clinicians completed the survey. Of the 17 participants identified as clients, 9 were young people and 8 were the parents of the young people who completed the survey. All participants completed the survey using the paper/post option, with none opting for the online survey.

#### Appointments attended

Ninety-one percent (91%) of professionals used Attend Anywhere for more than six appointments (*N* = 11). Fifty-three percent (53%) clients attended between three and six appointments (*N* = 9), thirty-five percent (35%) attended one to three appointments and twelve percent (12%) attended more than six (Supplementary Appendix S4).

#### Barriers prior to the introduction of attend anywhere

Table 2 outlines the frequencies for the two respondent groups for “Barriers prior to the introduction of Attend Anywhere”. Clients were asked to identify the barriers they experienced in accessing CAMHS. Professionals were asked to identify the barriers they thought clients experienced in accessing CAMHS. Separately, professionals were also asked to rate the barriers they themselves experienced in offering their services in CAMHS Clare. The overall frequency of reported barriers was *N* = 63. For professionals the most frequently reported barrier was accommodation issues (*N* = 11, 25%). Accommodation in this instance refers to clinical space used for meeting with clients and dedicated office space used for conducting clinical and other client-related work. For clients, time off school and time taken to travel to appointments were the most reported barriers (both *N* = 6, 32%).

**Table 2:** Frequency table for “Barriers prior to the introduction of Attend Anywhere” for the two respondent groups.

Variable	Professionals		Clients	
	*N*	%	*N*	%
Arranging transport	8	18.2	4	21.1
Time taken to travel to appointments	4	9.1	6	31.6
Time taken away from other activities	2	4.5	1	5.3
Time off school	10	22.7	6	31.6
Concerns re privacy/stigma	3	6.8	1	5.3
Feeling uncomfortable in CAMHS building	6	13.6	1	5.3
Accommodation (Professionals only)	11	25.0	–	–
Total	44	100%	19	100%

*N* refers to the number of times a response was selected by participants. % (percent) refers to the expressed value of *N* out of 100.

#### Barriers since the introduction of attend anywhere

Table 3 outlines the frequencies for the two respondent groups for “Barriers since the introduction of Attend Anywhere”. The overall frequency of reported barriers since the introduction of Attend Anywhere reduced by forty-three (*N* = 43) to twenty (*N* = 20). For professionals the most frequently reported barrier remained accommodation issues (*N* = 6, 46%). For clients the most reported barriers were arranging transport and time off school (both *N* = 2, 29%).

**Table 3:** Frequency table for “Barriers since the introduction of Attend Anywhere” for the two respondent groups.

Variable	Professionals		Clients	
	*N*	%	*N*	%
Arranging transport	1	7.7	2	28.6
Time taken to travel to appointments	0	0	1	14.3
Time taken away from other activities	1	7.7	1	14.3
Time off school	4	30.8	2	28.6
Concerns re privacy/stigma	1	7.7	0	0
Feeling uncomfortable in CAMHS building	0	0	1	14.3
Accommodation (Professionals only)	6	46.2	–	–
Total	13	100%	7	100%

*N* refers to the number of times a response was selected by participants. % (percent) refers to the expressed value of *N* out of 100.

#### Travel time

Fifty-three percent (53%) of clients reported a travel time of 0–20 min, while twenty-nine percent (29%) reported travel time of 40–60 min and eighteen percent (18%) reported travel times of 20–40 min. Professionals estimated that eighty-five percent (85%) of their clients travel between twenty and forty minutes to get to their appointments (Supplementary Appendix S5). Notably, client's reported travel time did not reduce after the introduction of Attend Anywhere.

#### Ease of use

Eighty-three percent (83%) of professionals agreed with the statement “I found Attend Anywhere Easy to Use” while sixty-five percent (65%) of clients also agreed that they found it easy to use (Supplementary Appendix S6).

#### Would use attend anywhere again

**Figure 1:**
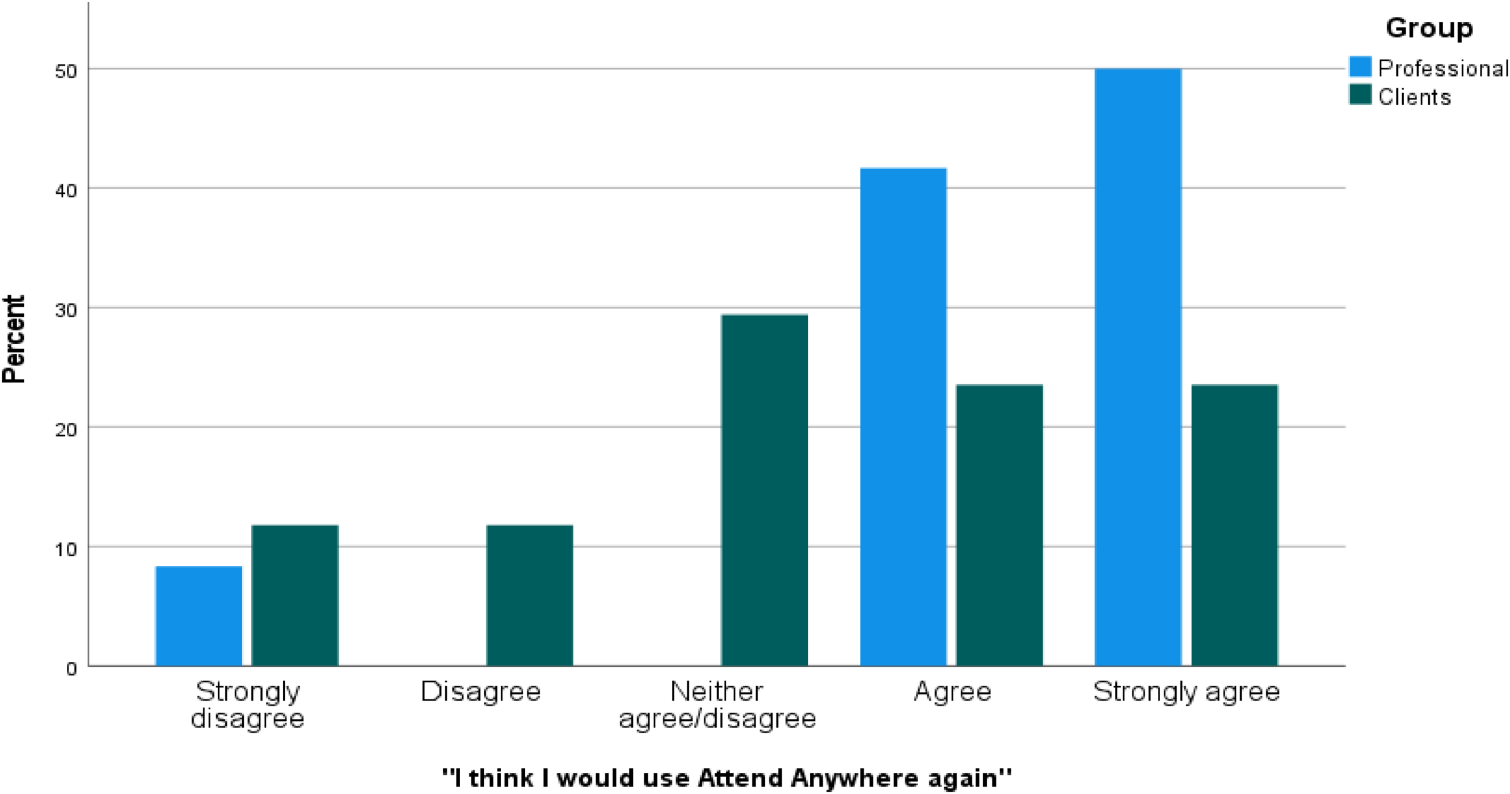
Percentage responses for “I think I would use Attend Anywhere again”. Percentage responses for “I think I would use Attend Anywhere again” categorised by group (Professional responses *N* = 12; Client responses *N* = 17), where N refers to the number of times a response was selected by participants and percent (%) refers to the expressed value of N out of 100.

Figure 1 outlines the percentage responses to the statement: “I think I would use Attend Anywhere again”. Over ninety percent (90%) of professionals agreed that they would use Attend Anywhere again (*N* = 11), while nearly fifty (50%) of clients also agreed (*N* = 8). An independent-samples t-test revealed that there was a significant difference in responses for professionals (M = 4.25, SD = 1.14) and clients [M = 3.18, SD = 1.42; *t*(29) = 2.165, *p* = .04, two-tailed], with professionals showing higher scores for agreeing to use Attend Anywhere again. The magnitude of the difference in the means [mean difference = 1.07, 95% CI (.06, 2.09)] was large [eta squared = 0.15; (17)]. The difference between clients and professionals in response to this statement is highlighted in Figure 1.

#### Benefits of using attend anywhere

Table 4 outlines the frequencies for the two respondent groups for “Benefits of using Attend Anywhere”. The most frequently reported benefit was ability to attend sessions from home for both professionals (*N* = 11, 31%) and clients (*N* = 13, 37%).

**Table 4:** Frequency table for “Benefits of using Attend Anywhere” for two respondent groups.

Variable	Professionals		Clients	
	*N*	%	*N*	%
Could attend from home	11	30.6	13	37.1
Could attend at a suitable time	8	22.2	7	20.0
Comfortable with video/technology	7	19.4	7	20.0
Felt more comfortable than in clinic	3	8.3	4	11.4
Did not need to rely on others to attend	7	19.4	4	11.4
Total	36	100%	35	100%

*N* refers to the number of times a response was selected by participants. % (percent) refers to the expressed value of *N* out of 100.

#### Participant dislikes in relation to attend anywhere

Connectivity issues were the highest reported aspect that both professionals and clients did not like (professionals *N* = 11, 34%; clients *N* = 7, 35%), followed by a preference for face-to-face work (professionals *N* = 7, 22%; clients *N* = 7, 35%). Professionals reported concerns regarding privacy (*N* = 5, 16%) while clients did not report any concerns with same. Clients reported that it was hard to develop the therapeutic relationship (*N* = 3, 15%) while professionals did not report any issues with this in survey item responses (Supplementary Appendix S7).

#### Ideal service provision going forward

**Figure 2:**
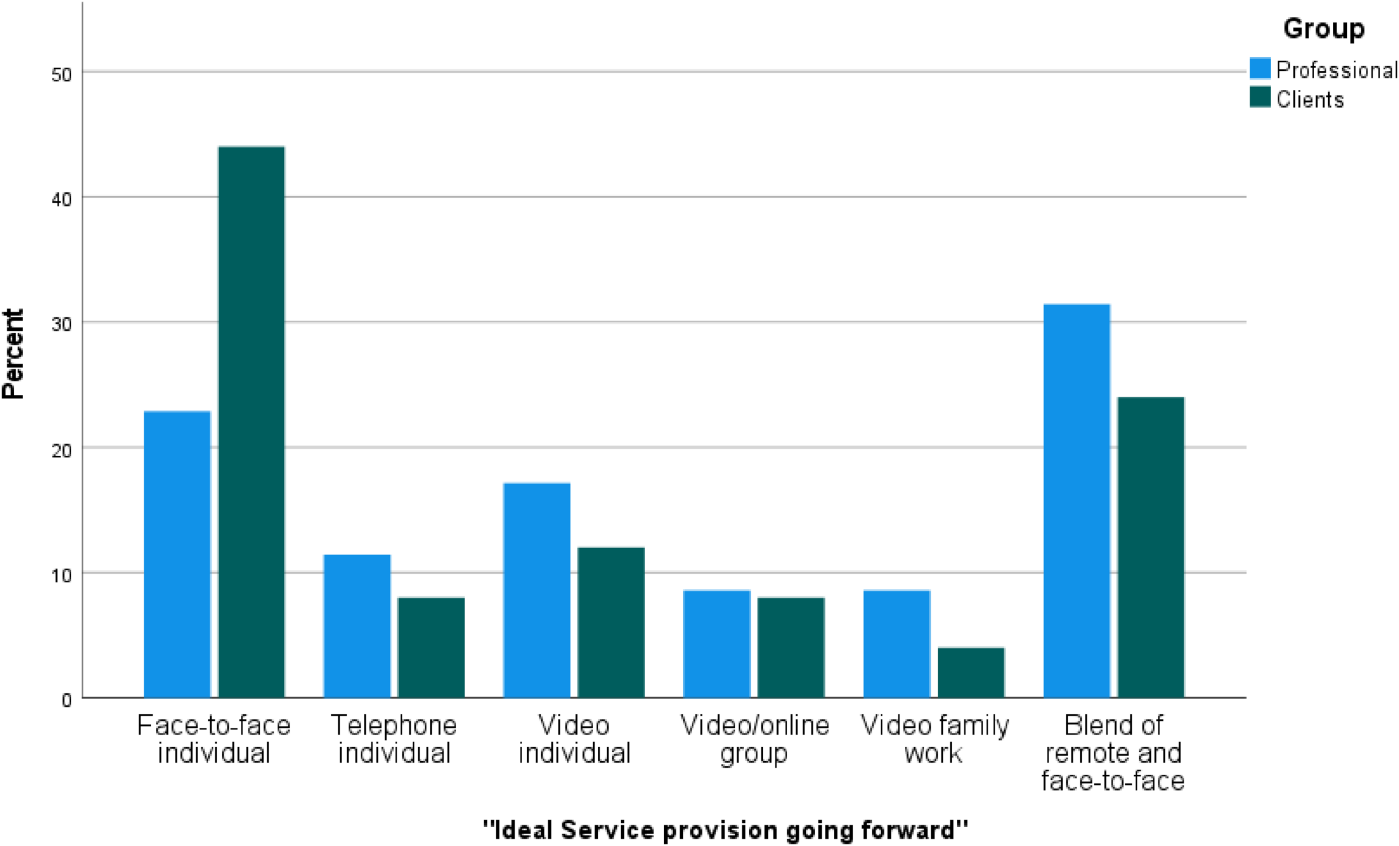
Percentage responses for “Ideal service provision going forward”. Percentage responses for “Ideal service provision going forward” categorised by group (Professional responses *N* = 35; Client responses *N* = 25), where *N* refers to the number of times a response was selected by participants and percent (%) refers to the expressed value of *N* out of 100.

Figure 2 outlines the percentage responses for “Ideal service provision going forward”. Professionals reported that ideally they would offer their CAMHS service through a blend of remote and face-to-face work (*N* = 11, 31%), followed by face-to-face individual work (*N* = 8, 22%). Clients reported a higher preference to receive their services via face-to-face individual work (*N* = 11, 44%), followed by a blend of remote work and face-to-face individual work (*N* = 6, 24%).

### Qualitative data analysis

Thematic Analysis was used to analyse qualitative data and to identify themes. Four major themes were identified: Improved Experience, Deficits and Concerns, Use of Technology, Appropriateness. For each of the themes, an overview will be provided, and reference will be made to the experiences of young people, their parents and health care professionals to separate the experiences of using Attend Anywhere for each group. Eight sub-themes were also identified which are presented along with the main themes in Table 5.

**Table 5:** Overview of themes and subthemes.

Major theme	Subtheme
Accessibility	Convenience Family Centred
Deficits and Concerns	Privacy Engagement Not a replacement
Use of Technology	Connectivity Disruption
Appropriateness	Clinical Age/Stage Preference

#### Accessibility

Participants reported on the ways in which using Attend Anywhere had improved their experience of accessing or working within CAMHS services. Young People identified convenience as the main improvement.

“I find it easy to use and more convenient”

Parents identified the benefits of accessing CAMHS using Attend Anywhere on family life. For example, when comparing Attend Anywhere sessions with attending face-to-face, parents noted the impact of travel to the clinic and parent involvement in sessions on everyday family routines.

“Other children needed to come out of school too for me to attend”

“It's more family friendly”

Professionals noted improvements in accessibility, in particular the ability for young people to connect from school, the ability for professionals to continue to see clients while working from home, and the use of Attend Anywhere to engage clients that may not have felt comfortable engaging with face-to-face intervention. Attend Anywhere was seen as a way of overcoming safety concerns due to COVID-19, clinical space limitations, and reduce disruption to school and family life. Professionals noted the use of Attend Anywhere within school settings in particular as a positive advancement in service provision.

“Beneficial when they can access sessions from school—less time missed”

“Attend Anywhere has been beneficial for young people with Social Anxiety and also young people who felt their home was safer and needed reassurance”

“AA helped them express and open up more and also allowed clinicians to provide intervention at child's own pace”

While some professionals noted the convenience of using Attend Anywhere to overcome accommodation or clinical space shortages, it was also noted that for clinicians sharing office spaces, the opportunities to use AA were limited due to the need to provide a confidential and uninterrupted space.

“Only a few clinical rooms to use, so cannot use own office to use Attend Anywhere if sharing with others”

#### Deficits and concerns

Overall, participants reported a number of deficits and concerns that arose in relation to engaging with the technology. These concerns focused on privacy and problems engaging in the therapeutic process for some young people and their parents. Across all groups, participants indicated that AA was not a suitable replacement for face-to-face intervention but a useful way of addressing short-term access barriers and a helpful addition to service provision for some.

Participants noted privacy concerns that arose while utilising AA at home. One young person noted:

“Family members present in the house could potentially overhear my conversations which I found unsettling”

Similarly, professionals noted the issue of privacy for the young person when connecting from home with their family present:

“Privacy for the child at times emerged. Parents would pass by and join and clinician would have to emphasize privacy”

Difficulties with engagement was highlighted most significantly by professionals. This group identified difficulties developing a relationship with a young person via computer particular where the young person was relatively new to CAMHS.

“Some challenges engaging young children in particular”

Interestingly, difficulties with regard to engagement were most commonly captured in the qualitative responses of professionals. This professional concern did not arise in the quantitative data.

Across all groups, AA was identified as a useful adjunct to service provision rather than a replacement. This was seen most often in the survey responses of professionals:

“May be useful for screening or follow-up appointments if appropriate initial assessment has been carried out. Not a replacement for an in-depth psychiatric assessment”

#### Use of technology

All professionals reported experiencing difficulty with regard to internet connectivity. Some parents also identified it as a challenge, but connectivity was not highlighted by young people in this study. Professionals and some parents noted the impact of disruptions due to technical issues on the session. Again, this was a concern highlighted by professionals and parents but not by young people.

One parent noted:

“Internet issues several times, screen freezing etc therapists got cut off”

Professional noted concerns with:

“poor connection at times”

“Connectivity issues, calls lagging / dropping etc.”

#### Appropriateness

The appropriateness of using Attend Anywhere as part of CAMHS service provision was mentioned by some professionals and parents in relation to clinical appropriateness, age appropriateness, the stage of the young person's journey with CAMHS, and individual preference.

Professionals in particular noted that Attend Anywhere was clinically appropriate for some presentations but not for others.

“Doesn’t suit all presentations”

Similarly, parents noted that face-to-face sessions were preferable when there were physical symptoms associated with their child's difficulty.

“It's easier using face to face meetings to interact with the therapist and they can observe physical difficulties my son is dealing with”

The stage at which the young person was at in terms of their engagement with CAMHS was also identified as important in considering appropriateness, particularly by professionals.

“It has been easier to use with young people where the relationship was already established”

The age of the young person was also highlighted as important to appropriateness of Attend Anywhere. Professionals indicated a preference for using it with an older age group of young people accessing CAMHS.

“Very useful for older teens”

Finally, across young people, parents and professional groups, the subtheme of preference arose. Professionals noted the preferences of young people and parents as a consideration separate to clinical and age appropriateness. While acknowledging the benefits that using Attend Anywhere may offer for young people and their families, one professional noted:

“Some young people and their families prefer face-to-face appointments”

Overall, age, stage of engagement, clinical considerations and preference were the main considerations identified across participants when discussing appropriateness of Attend Anywhere in a CAMHS setting.

## Discussion

This study aimed to explore the experiences of young people, parents and professionals of using Attend Anywhere video consultation service as part of their CAMHS service. The results demonstrate a decrease in the number of barriers reported by clients and professionals in accessing CAMHS following the introduction of Attend Anywhere in this service. Specifically, participants reported one-third less barriers to accessing their CAMHS service. The time taken to travel to appointments and the amount of time off school were the two biggest barriers to accessing face-to-face services for clients which were reduced by the implementation of Attend Anywhere. A major barrier reported by professionals in providing face-to-face intervention in this CAMHS service, was a shortage of clinical space. This may represent a substantial motivator for this particular group of professionals to adopt Attend Anywhere from the outset.

Both professionals and clients experienced difficulties in relation to connectivity issues, consistent with previous research findings (1, 2). Notably, clients still reported barriers in relation to arranging transport and time off school after Attend Anywhere was introduced. This may have been due to some clients needing to travel from school to home to attend video sessions.

Overall, the majority of professionals reported that they would use Attend Anywhere again as part of their mental health service, a finding consistent with previous research (1, 2). In contrast to previous findings, for clients the results were mixed: half of clients reported they would use it again, almost a quarter said they would not use it again while a third of clients neither agreed nor disagreed to use it again. Clients showed a strong preference for receiving face-to-face services over other service provision options. This finding was associated with not having to rely on technology, not having to manage connectivity issues and finding it easier to build the therapeutic relationship in person. Findings suggest that both professionals and clients see the value in face-to-face appointments and therefore, in line with Donaghy et al.'s (3) finding, suggest that VEC be used in conjunction with rather than as a replacement of face-to-face service provision. Attend Anywhere offers a way of providing services in a flexible manner which was particularly pertinent during the time period described.

In addition to overcoming clinical space shortages, the comparatively higher willingness of professionals in this study to adopt AA may reflect the supports and infrastructure put in place to train and facilitate professionals to use it. Prior to data collection commencing, the national rapid implementation of Attend Anywhere had begun and AA champions were in place across the region and were particularly active within this CAMHS service. The results of this study offer tentative support for the implementation of those structures put in place to support professionals during that time. The NHS Scotland evaluation of Attend Anywhere (2020) reported that organisations that adopted Attend Anywhere most readily had the following features: a receptive context for change (history of successful innovation; strong, visionary leaders; a clear, positive narrative about the technology; and good data systems that can monitor the effects of the change in a timely way), slack resources (people or money that could be channelled into the change effort), a predominance of supporters over opponents, and senior clinical and management buy-in. The NHS study concluded that variation in preconditions and the presence or absence of innovators, champions and change agents helped explain why different specialties and remote sites adopted Attend Anywhere at different rates.

Clients (young people and parents) in this study reported less of a willingness to use AA again when compared with professionals. It is also notable that professionals reported using AA more than the usage reported by clients. This may be reflective to an extent of the supports and infrastructure in place to facilitate adoption. While clients were directed to pre-recorded online guidance and received an information leaflet prior to their appointment, they did not have the formal interactive training or on-site support that professionals had access to. Additional information and training aimed at young people and parents may support further adoption of Attend Anywhere by clients accessing mental health services.

The hub-home model where the professional accesses AA from a specialist hub (the CAMHS clinic), and the client accesses from home, was the primary model used in this service. However, the qualitative data indicates the potential benefit of increased use of the triadic hub—spoke model where the client accesses AA from another setting (GP practise, school or other healthcare setting) and the professionals accesses from the specialist hub. In the current study, the triadic hub—spoke model facilitated clients to attend regular CAMHS appointments without impacting on functioning in other areas (for example attending AA from school with student counsellor support reduced school attendance issues). Future research exploring further implementation may be useful alongside an understanding of the training, technical infrastructure, clinical governance and operational guidance that may be required.

Limitations of this study are that the sample size is relatively small and there is a potential of response bias between those who completed the survey vs. those who did not. The study was conducted in one geographical area which limits the extent to which findings can be generalised. However, no research examining the experiences of young people, parents and professionals, of using Attend Anywhere in CAMHS is available to our knowledge. It was not possible to compare the responders to the remaining non-responders in terms of sample representativeness as no demographic information was available for the non-responders and we are unable to comment on the population as a whole. The survey was also completed anonymously eliminating the potential to identify the non-responder group and explore their experiences further. However, the findings from the participants who did respond maps out a pathway for future research in this area to explore the more nuanced aspects of using Attend Anywhere as part of service provision and provides us with good insight in terms of clients and professionals experiences using AA in this region. While the benefits of face-to-face service provision were outlined across all participant groups within the qualitative data collected, further quantitative data in relation to the benefits of face-to-face therapy for young people, parents and professionals would have allowed for more direct comparisons. As this was an anonymous survey, we were unable to analyse data in respect of participant age, diagnosis or other aspects of the young person's treatment. Similarly, we were unable to identify differences across professional disciplines. However, the anonymous nature of the study is likely to have increased engagement and participation. Future research would benefit from identifying which clinical presentations, age groups, and stages of engagement with CAMHS are most suited to using Attend Anywhere as well as what type of clinical inputs are most amenable to the use of AA.

Clients indicated a preference for receiving face-to-face services over other service provision options and this finding was associated with not having to rely on technology, manage connectivity issues, and finding it easier to build the therapeutic relationship in-person. Both clients and professionals valued face-to-face appointments, but clients indicated a stronger preference for face-to-face service provision than indicated by professionals in this study. Attend Anywhere reduced the barriers experienced in accessing CAMHS services in this region, and the majority of professionals wish to use it again going forward. The majority of clients (young people and parents) continue to prefer face-to-face service provision with 44% describing it as their ideal service going forward. One quarter of clients indicated that their ideal service would be a blend of face-to-face and VEC. We conclude that offering Attend Anywhere video consultation as part of a blended service along with face-to-face work can help to overcome access barriers. In the current study, Video Enabled Care was reported to reduce initial access barriers and in the provision of therapeutic intervention, clients indicated a preference for face-to-face services. Key considerations identified by participants in relation to the suitability of Attend Anywhere in this setting included: clinical appropriateness, age, stage of engagement with CAMHS, and personal preference.

## Data Availability

The raw data supporting the conclusions of this article will be made available by the authors, without undue reservation.

## References

[1] Houses of the Oireachtas Committee on the Future of Healthcare (2017). Sláintecare Report, May 2017. Houses of the Oireachtas: Dublin. http://hdl.handle.net/10147/622734 (Accessed January 27, 2023).

[2] Bradwell H Baines R Edwards KJ Stevens S Atkinson K Wilkinson E Exploring patient and staff experiences with video consultations during COVID-19 in an english outpatient care setting: secondary data analysis of routinely collected feedback data. JMIR Form Res. (2022) 6(3):e30486. 10.2196/30486 PMC8989384 35311688

[3] Donaghy E Atherton H Hammersley V McNeilly H Bikker A Robbins L Acceptability, benefits, and challenges of video consulting: a qualitative study in primary care. Br J Gen Pract. (2019) 69(686):e586–94. 10.3399/bjgp19X704141 31160368 PMC6617540

[4] Grindle KR. Impact of technology on community nursing during the pandemic. Br J Community Nurs. (2021) 26(3):110–5. 10.12968/bjcn.2021.26.3.110 33719559

[5] Isautier JM Copp T Ayre J Cvejic E Meyerowitz-Katz G Batcup C Lessons from the COVID-19 pandemic: people’s experiences and satisfaction with telehealth during the COVID-19 pandemic in Australia. *medRxiv*. (2020). 10.2196/24531 PMC7732356 33156806

[6] Johnstone-Burt A Giles S Wright R. P111. “Attend anywhere”: a virtual success? Patient feedback from breast nurse specialists using attend anywhere technology during the COVID-19 pandemic. Eur J Surg Oncol. (2021) 47(5):e325. 10.1016/j.ejso.2021.03.115

[7] Puttasiddaiah PM Morris S Dwamena S Sanu A. Attend anywhere clinic: a virtual outpatient clinic experience in otolaryngology during the COVID-19 pandemic. Laryngoscope Investig Otolaryngol. (2021) 6(3):586–9. 10.1002/lio2.557. 34195381 PMC8223447

[8] Lane A Clarke V. Report on the Findings of the First National Evaluation of the Use of Video Enabled Care in Ireland. Health Service Executive, Dublin (2021). Available at: https://healthservice.hse.ie/filelibrary/onmsd/report-on-the-findings-of-the-first-national-evaluation-of-the-use-of-video-enabled-health-care-in-ireland.pdf (Accessed December 30, 2023).

[9] Hammersley V Donaghy E Parker R McNeilly H Atherton H Bikker A Comparing the content and quality of video, telephone, and face-to-face consultations: a non-randomised, quasi-experimental, exploratory study in UK primary care. Br J Gen Pract. (2019) 69(686):e595–604. 10.3399/bjgp19X704573 31262846 PMC6607843

[10] Nouri S Khoong EC Lyles CR Karliner L. Addressing equity in telemedicine for chronic disease management during the COVID-19 pandemic. NEJM Catal Innov Care Deliv. (2020). Available at: https://catalyst.nejm.org/doi/full/10.1056/CAT.20.0123

[11] Wykes T Lipshitz J Schueller SM. Towards the design of ethical standards related to digital mental health and all its applications. Curr Treat Options Psych. (2019) 6:232–42. 10.1007/s40501-019-00180-0

[12] Wies B Landers C Lenca M. Digital mental health for young people: a scoping review of ethical promises and challenges. Front Digit Health. (2021) 3:697072. 10.3389/fdgth.2021.697072 34713173 PMC8521997

[13] Lundy L. “Voice” is not enough: conceptualising article 12 of the united nations convention on the rights of the child. Br Educ Res J. (2007) 33(6):927–42. 10.1080/01411920701657033

[14] Department of Children, Equality, Disability, Integration and Youth. National Strategy on Children and Young People's Participation in Decision-Making. (2019). Available at: https://www.gov.ie/en/publication/9128db-national-strategy-on-childrenand-young-peoples-participation-in-dec/ (Accessed October 12, 2022).

[15] Wherton & Greenhalgh. Evaluation of the Attend Anywhere / Near Me video consulting service in Scotland, 2019-20. University of Oxford. (2020). Available at: https://www.gov.scot/binaries/content/documents/govscot/publications/research-and-analysis/2020/07/evaluation-attend-anywhere-near-video-consulting-service-scotland-2019-20-main-report/documents/evaluation-attend-anywhere-near-video-consulting-service-scotland-2019-20/evaluation-attend-anywhere-near-video-consulting-service-scotland-2019-20/govscot%3Adocument/evaluation-attend-anywhere-near-video-consulting-service-scotland-2019-20.pdf (Accessed November 11, 2022).

[16] Braun V Clarke V. Using thematic analyses in psychology. Qual. Res. Psychol. (2006) 3:77–101.

[17] Pallant J. SPSS Survival manual: A step by step guide to data analysis using IBM SPSS. New York: Routledge (2020).

