# Supplementary material for: The experiences of young people, parents and professionals of using the attend anywhere video consultation system in a child and adolescent mental health service: a mixed-methods approach
Source: Front Child Adolesc Psychiatry. 2023 Aug 11;2:1194302. doi: 10.3389/frcha.2023.1194302 (PMC11731611; doi:10.3389/frcha.2023.1194302)
Supplement: Supplementary file 1 [file Datasheet1.docx]

**APPENDICES**

**Appendix 1: Attend Anywhere Survey**

**Title: Experiences of Using the Attend Anywhere Video Consulting Service in CAMHS: perspectives of young people, parents and professionals.**

I am a Young Person Parent Professional

1. How many Attend Anywhere Video-based appointments have you facilitated approximately?

1 – 3 3 - 6 More than 6

1. Prior to the introduction of Attend Anywhere, what barriers or challenges did you experience in facilitating face-to-face appointments in CAMHS? *(Please place a tick beside the challenges(s) that you experienced)*

Accommodation/space issues (e.g. accessibility of clinic rooms)

Client transport needs

Time taken for client to travel to the CAMHS building

Time away from young person’s hobbies / interests / social activities

Young person’s time off school

Client concerns about privacy or stigma (i.e. being seen by a peer in a CAMHS building)

Client feeling uncomfortable in the CAMHS building

Other (please add any other barriers to attending CAMHS for face-to-face appointments):

3. Since the introduction of Attend Anywhere what barriers or challenges did you experience? *(Please place a tick beside the challenge(s) you experience since the introduction of Attend Anywhere)*

Accommodation/space issues (e.g. accessibility of clinic rooms)

Client transport needs

Time taken for client to travel to the CAMHS building

Time away from young person’s hobbies / interests / social activities

Young person’s time off school

Client concerns about privacy or stigma (i.e. being seen by a peer in a CAMHS building)

Client feeling uncomfortable in the CAMHS building

Other (please add any other barriers to attending CAMHS for face-to-face appointments).

4. On average, how much time in total does it take for your clients to travel to attend one face-to-face appointment in CAMHS? *(Please tick the answer that applies to you)*

0 - 20 mins 20 - 40 mins 40 - 60 mins Over an hour

5. Please score the following statements using the scale provided below:

"I thought the Attend Anywhere Video Consulting System was easy to use"

Strongly disagree 1

Disagree 2

Neither agree nor disagree 3

Agree 4

Strongly agree 5

6. "I think that I would use Attend Anywhere again"

Strongly disagree 1

Disagree 2

Neither agree nor disagree 3

Agree 4

Strongly agree 5

7. What were the benefits of using Attend Anywhere? (*Please place a tick beside the statement(s) that are true for you)*

I could facilitate appointments from home/remotely

I could facilitate appointments at a time that suited the client

I am comfortable with using video calls and other forms of digital technology to access services

It was more comfortable for the client than in a clinical environment

The young person did not need to rely on others to ensure attendance at their session

Other: please describe the benefits or positive impacts (if any) of using Attend Anywhere for you.

8. What did you not like about using Attend Anywhere?

I found Attend Anywhere difficult to use

I experienced internet connectivity problems

I prefer meeting with clients face-to-face

I prefer phone rather than video contact with clients

I find it hard to develop a relationship with clients by video

I had concerns about privacy

I experienced problems with the device I was using

Other (please add any other concerns you have about using Attend Anywhere here).

9. Please add any other information about your experience of using Attend Anywhere that you wish to share here.

10. Ideally, going forward what ways of providing a CAMHS service would you be comfortable with?

Face-to-face individual work

Telephone-based individual work

Video-based individual work

Video or online-based group intervention

Video-based family work

A blend of remote and face-to-face work

**Appendix 2: Information Sheet**

**Participant Information Sheet (Young Person & Parent/Guardian)**

Attend Anywhere has been introduced in CAMHS Clare as a way for young people and their parents to have their appointments with CAMHS professionals by video. As this is a new way of providing services, we are interested in finding out about the experiences of young people and their parents who have used it. This information would help us to plan how we provide services going forward.

We are asking young people and their parents who consent to taking part, to complete a brief survey about their experiences. We estimate that the survey will take 5-7 minutes to complete in total.

The survey can be completed on **paper.** We have enclosed a copy of the survey, consent form, and stamped addressed envelope.

Alternatively the survey can be complete **online** using the URL below:

<https://www.surveymonkey.com/r/RD3KJC3>

**Appendix 3: Scatterplot for “I would Use Attend Anywhere again” variable**


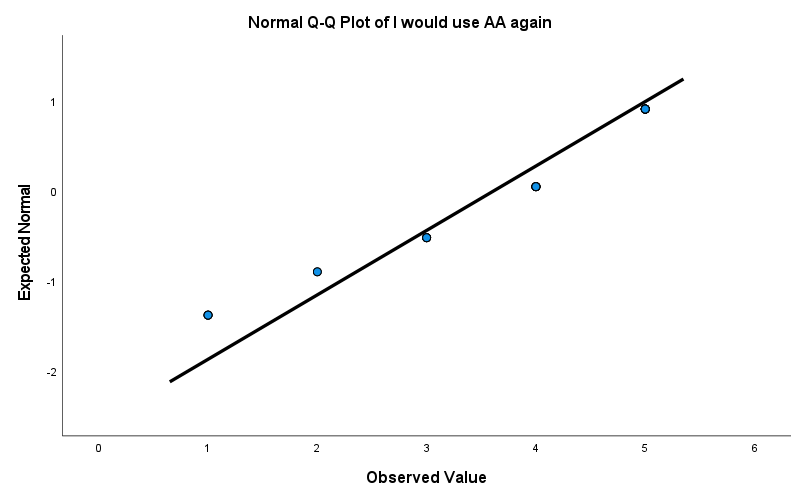


* A Normal Q-Q plot used to assess if data is normally distributed. If data is normally distributed, the points in a Q-Q plot will lie on a straight diagonal line.

**Appendix 4: Appointments Attended**

**
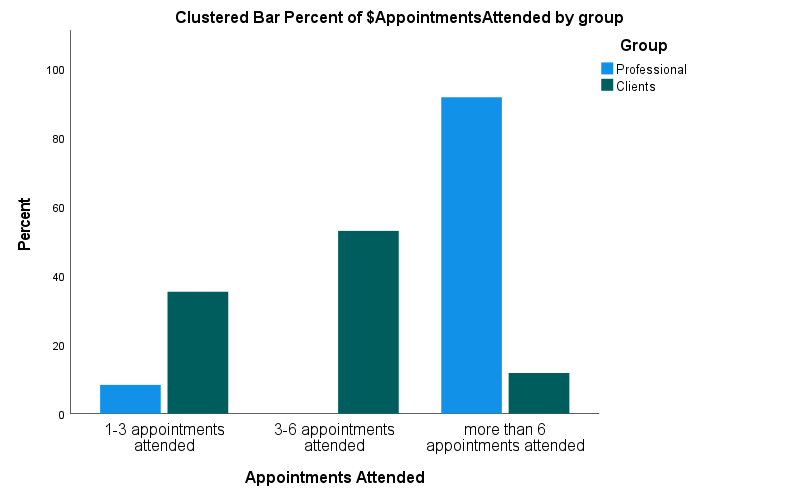
**

**Appendix 5: Travel Time**


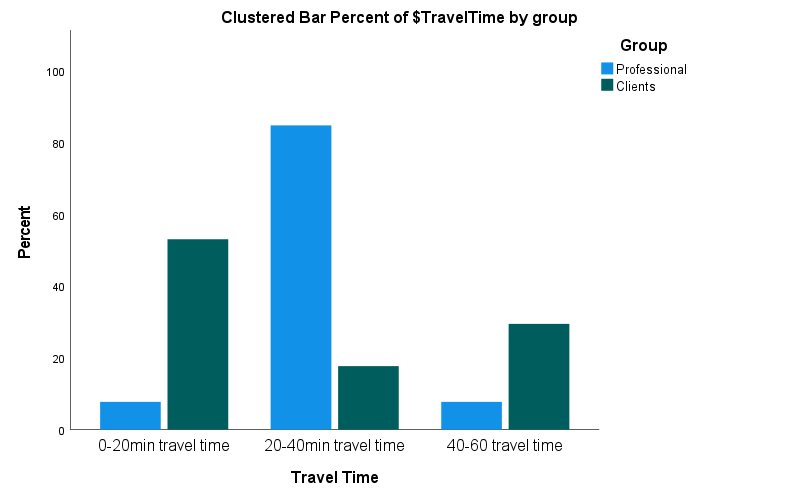


**Appendix 6: Easy to use**


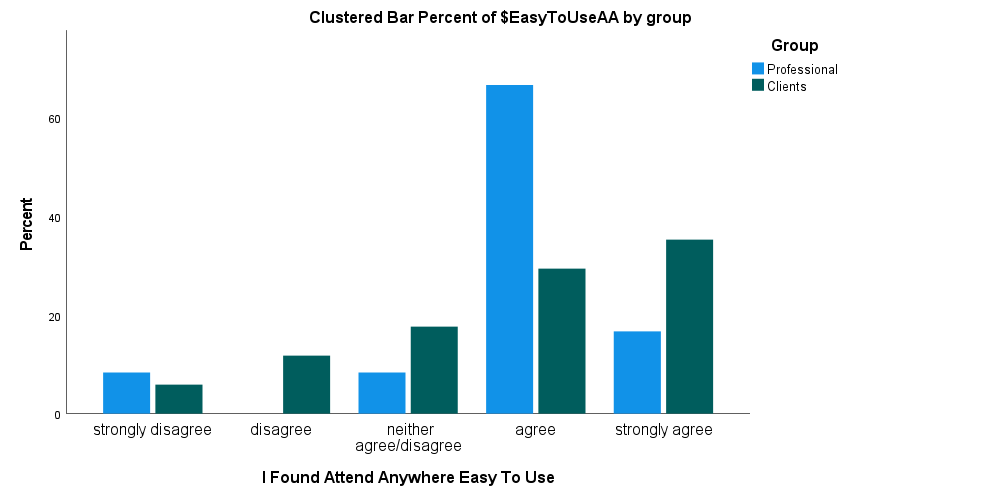


**Appendix 7: Participant Dislikes**

**
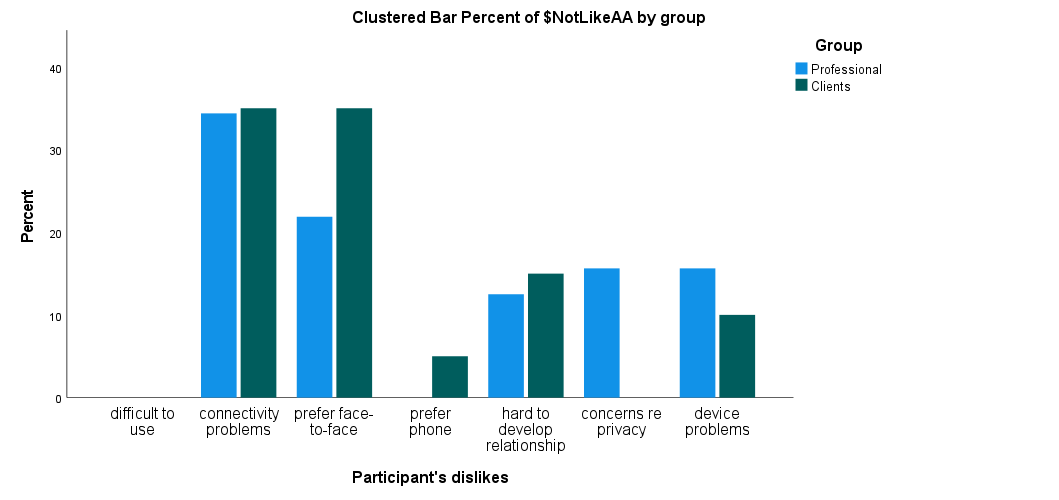
**
